# Is It Time for Pediatric and Adult Gastroenterologists to Assume Responsibility for Providing Vaccinations?

**DOI:** 10.1093/crocol/otaa057

**Published:** 2020-07-07

**Authors:** Stacey Rolak, Freddy Caldera

**Affiliations:** 1 Department of Medicine, University of Wisconsin—Madison, School of Medicine and Public Health, Madison, Wisconsin; 2 Department of Medicine, Division of Gastroenterology and Hepatology, University of Wisconsin—Madison, School of Medicine and Public Health, Madison, Wisconsin

The incidence of inflammatory bowel disease (IBD) among children continues to rise in North America.^1^ Advances in the treatment of pediatric IBD have seen an introduction of novel immunosuppressive therapies, such as tumor necrosis factor (TNF) alpha inhibitors and anti-integrin agents. These agents achieve higher rates of clinical remission and mucosal healing than conventional therapy, but are associated with an increased risk for infections, including vaccine-preventable diseases.^2^ This well-established heightened risk of infection can be mitigated through the administration of routine childhood immunizations, and there are clear guidelines recommending that immunosuppressed children to follow the same routine immunization schedule as in healthy children.^2^ Despite this, vaccination coverage in pediatric patients with IBD is suboptimal and often lower than healthy children.^3^ The reasons for vaccine refusal among adults with IBD are well described^4^; however, the reasons underlying reduced vaccine uptake in pediatric populations have been less clearly elucidated. The study “Vaccination Rates and Family Barriers Among Children with Inflammatory Bowel Disease” examines the reasons for vaccine hesitancy among caregivers of children with IBD.

The study by Holland et al published in this volume of *Crohn’s & Colitis 360* was a single-center cross-sectional study of vaccine attitudes of caregivers and vaccination status among patients being followed in a pediatric gastroenterology clinic.^5^ One hundred and eight caregivers of children ages 10–25 years old were surveyed with validated questions regarding vaccine safety and opinions. Patient vaccination status was obtained from a statewide registry. The study sample consisted of 50 families with a child with IBD, and a control group of 58 families with a child being followed for a non-IBD gastrointestinal complaint. There was no difference in the uptake of the routine immunizations between the 2 groups; the only difference was that those with IBD had greater rates of Hepatitis B vaccination. Interestingly, this study showed that 1) caregivers of children with IBD were more concerned that vaccines would worsen their child’s underlying disease and 2) caregivers of patients with IBD were more concerned that vaccines are ineffective when compared to non-IBD caregivers.

The results of this study suggest an insufficient understanding of the impacts of vaccination on immunosuppressed pediatric patients among their caregivers. There are no data to suggest that vaccinations lead to disease flares in pediatric or adult patients with IBD; additionally, vaccinations are effective in patients with IBD, with some exceptions among patients on specific immunosuppressant regimens.^6–9^ One multicenter prospective controlled trial evaluating the immunogenicity of a tetanus, diphtheria, and acellular pertussis booster found that vaccine responses were similar between pediatric patients with IBD receiving no immunosuppressive therapy, those on thiopurines only, those on combination therapy (thiopurines and anti-TNF-α agents), and healthy controls.^6^ A similar trial found no significant difference in the immunogenicity of the 13-valent pneumococcal conjugate vaccine between pediatric patients with IBD and healthy controls, though patients with IBD on no immunosuppressive therapy did have higher postvaccination titer rates than did IBD patients on anti-TNF agents or immunomodulators.^7^ Studies evaluating the immunogenicity of vaccines in adult patients with IBD have also found that those on aminosalicylate or thiopurine monotherapy can mount normal vaccine responses similarly to healthy controls.^9^ Those on anti-TNF agents, especially those used in combination therapy with a thiopurine or methotrexate, may have a blunted vaccine response to certain vaccines.^9^

Additionally, there is no evidence to suggest that immunizations are associated with flares of IBD. No studies in pediatric or adult patients have reported significant increases in disease flares or serious systemic adverse effects following vaccination.^6–9^ A recent study examining the impact of the new recombinant herpes zoster vaccine, which has a novel new adjuvant, on adult patients with IBD showed no significant increase in disease flares, or local or systemic adverse effects following immunization.^10^ Despite this, fear of IBD flares is an underlying reason for vaccine hesitancy among both pediatric and adult patients with IBD.^4, 11^ One study found that among adult patients with IBD, those who declined the influenza vaccine were significantly more concerned about side effects, effectiveness, and the worsening of their IBD by vaccines.^4^ Patients and caregivers are probably unaware that there is no evidence to support these fears. Dispelling these misconceptions about vaccines will be critical in improving vaccination rates, which is especially important considering that patients with IBD are at a heightened risk for infections, due to both their underlying disease and as a complication of immunosuppressive therapy. It is important for families to realize that vaccines are both effective and safe for their children with IBD, and gastroenterologists have a responsibility to improve patient understanding on this topic.

Although primary care providers traditionally counsel and ensure that patients are up to date on their immunizations, they may be uncomfortable with vaccine counseling for immunosuppressed patients with IBD. In one survey of outpatient family medicine physicians, only 37% felt comfortable providing routine preventive care for their patients with IBD, and many reported discomfort providing vaccinations for this population.^12^ There has been a lack of clarity among patients and providers about who is responsible for vaccine counseling. One study found that provider recommendation was one of the most frequently cited motivations by patients with IBD for receiving the influenza vaccine. Just under half of the study population placed the responsibility of vaccine health maintenance on their gastroenterologist, whereas 62.1% placed it on their primary care physician.^4^ The results of the study by Holland et al showed similar opinions, with many families believing that pediatric specialists should discuss vaccines along with the primary care provider.^5^ This highlights that patients expect and appreciate input from their gastroenterologist on vaccinations. Clinical guidelines suggest that specialists share the responsibility of ensuring proper vaccination status among immunosuppressed patients with primary care providers.^13^ Despite this, the discrepancy about who should take primary responsibility remains.

To improve immunization status in patients, pediatric and adult gastroenterologists should assume or share the responsibility for immunizations with other providers. This point is particularly salient now, during the COVID-19 pandemic. Significantly fewer primary care and specialty physician visits are occurring in-person as more institutions adopt telemedicine appointments. This has already resulted in a decline in vaccination administration rates across the United States.^14^ This is especially worrisome as patients with IBD are already at an increased risk for many vaccine-preventable diseases; the inability to access routine immunizations further compounds their risk. Efforts should be made to provide vaccinations to patients who are seen at in-person gastroenterology visits or at other ancillary appointments, rather than assuming the patient will be able to receive vaccinations at their primary care office in a timely manner. Additionally, it is paramount to make education on the safety and efficacy of vaccines a priority now, so that patients with IBD are not deterred from receiving a COVID-19 vaccine once one becomes available in the future.

Efforts to educate patients and families about the importance and safety of vaccines have been beneficial in improving vaccination rates.^11, 15^ In addition to providing regular counseling, vaccines should be available to be administered in clinic. The physical presence of a vaccine in clinic improves both the reported prospective likelihood of a parent accepting a vaccination and the actual administration of vaccines, and improves convenience for parents.^11^ In conclusion, pediatric and adult gastroenterologists should take an active role in health maintenance by screening their patients at the time of diagnosis, regularly educating families on the benefits of vaccines, assuaging concerns about vaccine efficacy and worsening of disease, and administering vaccines in clinic.

## References

[1] Benchimol EI , Bernstein CN, Bitton A, et al Trends in epidemiology of pediatric inflammatory bowel disease in Canada: distributed network analysis of multiple population-based provincial health administrative databases. Am J Gastroenterol. 2017;112:1120–1134. 28417994 10.1038/ajg.2017.97 PMC5527278

[2] Ardura MI , Toussi SS, Siegel JD, et al NASPGHAN clinical report: surveillance, diagnosis, and prevention of infectious diseases in pediatric patients with inflammatory bowel disease receiving tumor necrosis factor-α inhibitors. J Pediatr Gastroenterol Nutr. 2016;63:130–155. 27027903 10.1097/MPG.0000000000001188

[3] Dipasquale V , Romano C. Vaccination strategies in pediatric inflammatory bowel disease. Vaccine. 2017;35:6070–6075. 28967524 10.1016/j.vaccine.2017.09.031

[4] Wasan SK , Calderwood AH, Long MD, et al Immunization rates and vaccine beliefs among patients with inflammatory bowel disease: an opportunity for improvement. Inflamm Bowel Dis. 2014;20:246–250. 24374881 10.1097/01.MIB.0000437737.68841.87 PMC4393851

[5] Holland KJ , Wilkinson TA, Phipps E, et al Vaccination rates and family barriers among children with inflammatory bowel disease. Crohns Colitis 360. 2020. [Epub ahead of print] 10.1093/crocol/otaa056 PMC8015385 33817638

[6] Banaszkiewicz A , Gawronska A, Klincewicz B, et al Immunogenicity of pertussis booster vaccination in children and adolescents with inflammatory bowel disease. Inflamm Bowel Dis. 2017;23:847–852. 28394806 10.1097/MIB.0000000000001076

[7] Banaszkiewicz A , Targońska B, Kowalska-Duplaga K, et al Immunogenicity of 13-valent pneumococcal conjugate vaccine in pediatric patients with inflammatory bowel disease. Inflamm Bowel Dis. 2015;21:1607–1614. 25919976 10.1097/MIB.0000000000000406

[8] Caldera F , Hillman L, Saha S, et al Immunogenicity of high dose influenza vaccine for patients with inflammatory bowel disease on anti-TNF monotherapy: a randomized clinical trial. Inflamm Bowel Dis. 2020;26:593–602. 31504526 10.1093/ibd/izz164

[9] Nguyen DL , Nguyen ET, Bechtold ML. Effect of immunosuppressive therapies for the treatment of inflammatory bowel disease on response to routine vaccinations: a meta-analysis. Dig Dis Sci. 2015;60:2446–2453. 25796579 10.1007/s10620-015-3631-y

[10] Satyam VR , Li P-H, Reich J, et al Safety of recombinant zoster vaccine in patients with inflammatory bowel disease. Dig Dis Sci. 2020. [Epub ahead of print] doi:10.1007/s10620-019-06016-4 31897892

[11] Huth K , Benchimol EI, Aglipay M, et al Strategies to improve influenza vaccination in pediatric inflammatory bowel disease through education and access. Inflamm Bowel Dis. 2015;21:1761–1768. 26199989 10.1097/MIB.0000000000000425

[12] Selby L , Hoellein A, Wilson JF. Are primary care providers uncomfortable providing routine preventive care for inflammatory bowel disease patients? Dig Dis Sci. 2011;56:819–824. 20668942 10.1007/s10620-010-1329-8

[13] Rubin LG , Levin MJ, Ljungman P, et al Executive summary: 2013 IDSA clinical practice guideline for vaccination of the immunocompromised host. Clin Infect Dis. 2014;58:309–318. 24421306 10.1093/cid/cit816

[14] Santoli JM , Lindley MC, Desilva MB, et al Effects of the COVID-19 pandemic on routine pediatric vaccine ordering and administration – United States, 2020. MorbMortal Wkly Rep. 2020;69:591–593. 10.15585/mmwr.mm6919e2 32407298

[15] Fleurier A , Pelatan C, Willot S, et al Vaccination coverage of children with inflammatory bowel disease after an awareness campaign on the risk of infection. Dig Liver Dis. 2015;47:460–464. 25770456 10.1016/j.dld.2015.02.009

